# P-552. Viral load non-suppression among adults living with HIV on antiretroviral treatment in Sughd Region, Tajikistan, 2013-2022

**DOI:** 10.1093/ofid/ofae631.751

**Published:** 2025-01-29

**Authors:** Emomali Qurbonov, Dilyara Nabirova, Roberta Horth, Aisuluu Kubatova

**Affiliations:** Central Asia Advanced Field Epidemiology Training Program, Khujand, Sughd, Tajikistan; CDC Central Asia office, Almaty, Almaty, Kazakhstan; US Centers for Disease Control and Prevention, Dulles, Virginia; Ministry of Health of the Kyrgyz Republic, National Institute of Public Health, Bishkek, Kyrgyzstan, Bishkek, Bishkek, Kyrgyzstan

## Abstract

**Background:**

Unlike many other regions, Central Asia has an increasing incidence of HIV. Viral load suppression among people living with HIV (PLHIV) is a key strategy for reducing HIV transmission. We conducted a study to identify factors associated with viral load non-suppression among PLHIV on antiretroviral treatment (ART).

**Figure:**
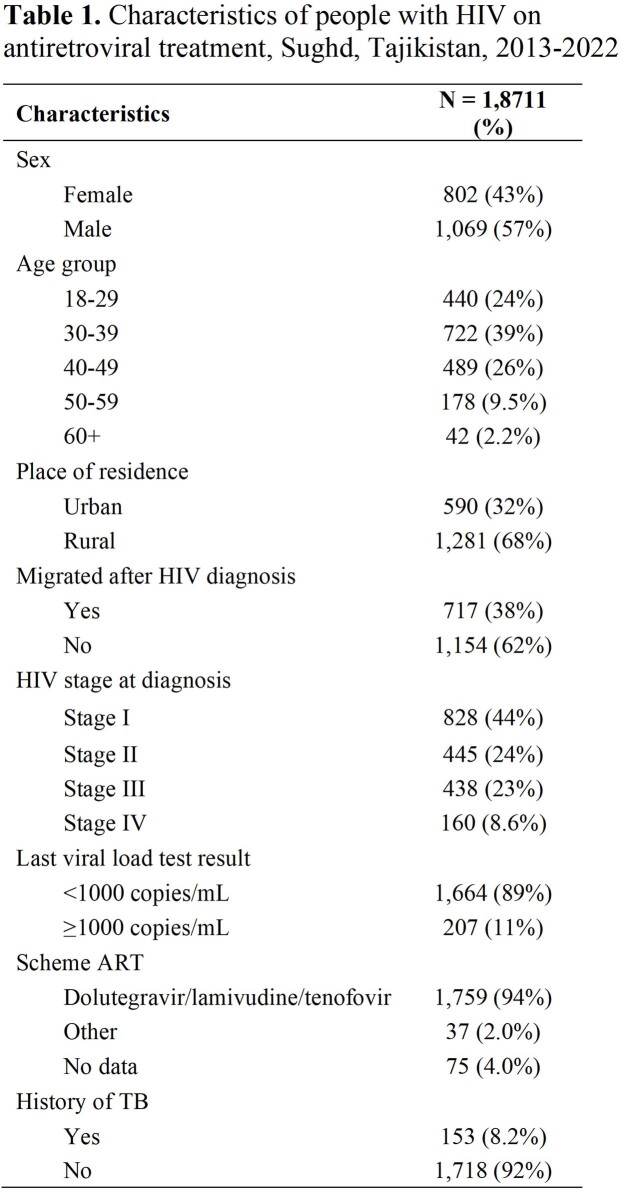

**Methods:**

We conducted a retrospective study of adults (≥18 years old) newly diagnosed with HIV from 2013 to 2022 who received ART for 6+ months in the Sughd region. Data were abstracted from the national electronic registry of PLHIV and cross-checked with paper medical and laboratory records. Viral load non-suppression was defined as anyone with >1000 copies per ml after being on 6+ months of treatment. Descriptive statistics were performed to summarize the characteristics of the study participants. Bivariable and multivariable logistic regression was used to identify factors associated with viral load non-suppression. We present adjusted odds ratios (aOR) and 95% confidence intervals (95%CI).

**Table 2.**
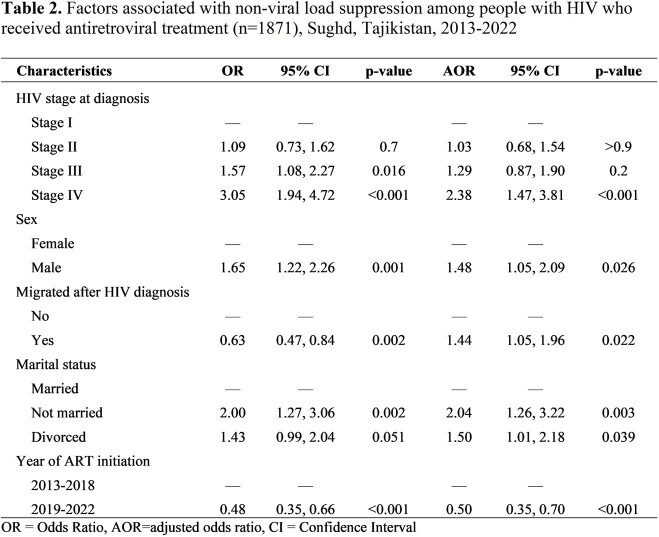
Factors associated with non-viral load suppression among adults with HIV who received antiretroviral treatment (n=1871), Sughd, Tajikistan, 2013-2022

**Results:**

Of 1,871 people who were on ART for at least 6 months from 2013 to 2022, 11% did not achieve viral suppression. Of people on ART, 57% were male, 38% were migrants, and 68% lived in a rural area. The mean age was 31 years (range: 18-74) and 8% had a history of TB. One-third (32%) had late HIV diagnosis (23% in stage 3 and 9% in stage 4). The majority (94%) were on tenofovir lamivudine/dolutegravir (TDF/3TC/DTG) **(Table 1).** People diagnosed with stage 4 disease vs stage 1 (aOR=2.4, 95%CI=1.5–3.8; p< 0.01), males vs females (aOR=1.5, 95%CI=1.1-2.0; p=0.03), people who migrated after HIV diagnosis vs non-migrants (aOR=1.4, 95%CI=1.1-2.0; p=0.02), or were not married (single) vs married (aOR = 2.0, 95%CI=1.3–3.2; p=< 0.01) had increased odds of viral load non-suppression. People diagnosed in 2019-2022 had lower odds of viral load non-suppression compared to people diagnosed in 2013-2018 (aOR=0.5, 95%CI=0.4-0.7; p< 0.01) **(Table 2).**

**Conclusion:**

Level of viral load suppression among people on ART in Sughd region of Tajikistan is below the global target of 95%. Increasing early detection and providing treatment support for groups with higher odds of non-suppression, especially males, migrants and people in later stages of the disease, can help with achieving global targets.

**Disclosures:**

All Authors: No reported disclosures

